# INDIVIDUAL AND SOCIAL FACILITATORS OF PHYSICAL ACTIVITY IN STROKE SURVIVORS: EVIDENCE FROM DAILY LIFE ASSESSMENTS

**DOI:** 10.1093/geroni/igae098.4223

**Published:** 2024-12-31

**Authors:** Elizabeth Zambrano Garza, Theresa Pauly, Rachel Murphy, Maureen Ashe, Kenneth Madden, Wolfgang Linden, Denis Gerstorf, Christiane Hoppmann

**Affiliations:** University of British Columbia, Vancouver, British Columbia, Canada; Simon Fraser University, Vancouver, British Columbia, Canada; University of British Columbia, Vancouver, British Columbia, Canada; University of British Columbia, Vancouver, British Columbia, Canada; University of British Columbia, Vancouver, British Columbia, Canada; University of British Columbia, Vancouver, British Columbia, Canada; Humboldt University Berlin, Berlin, Berlin, Germany; University of British Columbia, Vancouver, British Columbia, Canada

## Abstract

Physical activity is a means through which stroke survivors can reduce their future stroke risk. Individual intentions (purposeful aims to engage in a behavior) and self-efficacy (beliefs about their capacity to successfully perform the behavior) have been associated with more physical activity. Spouses are underrecognized resources that may facilitate physical activity engagement. We specifically examined dyadic intentions (shared aims to engage in a behavior) and dyadic self-efficacy (beliefs about the capacity to engage in a behavior with a partner) to explore the important role of spouses in facilitating physical activity. 88 Canadian stroke survivors (Mage = 67.84, SD = 10.79; 73% male; 35% with college degrees; 82% White) wore physical activity monitors for up to 14 consecutive days and completed morning and evening questionnaires including daily individual and dyadic intentions and self-efficacy. Results reveal positive associations between individual morning intentions and physical activity (self-report and accelerometer based) but not for individual self-efficacy. Dyadic variables (intentions and self-efficacy) were positively associated with self-reported physical activity but not with accelerometer assessed physical activity. Notably, associations differed by analytical level: within-person effects were significant for individual intentions and dyadic self-efficacy, whereas between-person effects were significant for both individual and dyadic intentions. This project highlights and discusses the importance of considering within and between person effect to provide more granular insights on the complexity of the targeted phenomenon as well as issues of physical activity measurement (self-report, device-derived) to light on avenues for increasing physical activity engagement in stroke survivors.

